# Association of the Yips and Musculoskeletal Problems in Highly Skilled Golfers: A Large Scale Epidemiological Study in Japan

**DOI:** 10.3390/sports9060071

**Published:** 2021-05-21

**Authors:** Yasufumi Gon, Daijiro Kabata, Sadahito Kawamura, Masahito Mihara, Ayumi Shintani, Ken Nakata, Hideki Mochizuki

**Affiliations:** 1 Department of Neurology, Graduate School of Medicine, Osaka University, 2-2 Yamada-oka, Suita, Osaka 565-0871, Japan; hmochizuki@neurol.med.osaka-u.ac.jp; 2 Department of Medical Statistics, Graduate School of Medicine, Osaka City University, 1-4-3 Asahi-machi, Abeno-ku, Osaka 545-8586, Japan; kabata.daijiro@med.osaka-cu.ac.jp (D.K.); ayumi.shintani@gmail.com (A.S.); 3 Department of Medicine for Sports and Performing Arts, Graduate School of Medicine, Osaka University, 2-2 Yamada-oka, Suita, Osaka 565-0871, Japan; kawamura@bc.iij4u.or.jp (S.K.); ken.nakata7@gmail.com (K.N.); 4 Kawamura Orthopedic Clinic, 1-10-30 Misaki, Minato-ku, Osaka 552-0016, Japan; 5 Department of Neurology, Kawasaki Medical University, 557 Matsushima, Kurashiki, Okayama 701-0192, Japan; mihara@med.kawasaki-m.ac.jp

**Keywords:** yips, golfing career, musculoskeletal symptoms, task-specific dystonia

## Abstract

The yips are a set of conditions associated with intermittent motor disturbances that affect precision movement, especially in sports. Specifically, skilled golfers suffer from the yips, although its clinical characteristics and pathophysiology have not been well-studied. We surveyed skilled golfers to characterize their yips-related symptoms, to explore potential confounding factors associated with the yips. Golfers’ demographic information, golfing-career-related history, musculoskeletal status and manifestations of the yips are surveyed. Among the 1576 questionnaires distributed, 1457 (92%) responses were received, of which 39% of golfers had experienced the yips. The median age and golfing careers were 48 and 28 years, respectively. Golfers who had experienced the yips were older and had longer golfing careers and more frequent musculoskeletal problems than those without experience of the yips. The multivariate logistic regression analysis revealed that a longer golfing career and musculoskeletal problems were independent factors associated with yips experience. More severe musculoskeletal problems were associated with higher odds of experiencing the yips. A positive association between the yips and musculoskeletal problems was also observed. The yips have similar characteristics to task-specific movement disorders, with a detrimental effect caused by excessive repetition of a routine task. These findings support the notion that the yips are a type of task-specific dystonia.

## 1. Introduction

Physical practice is essential for the acquisition and maintenance of skilled movements in humans. However, prolonged practice may lead to maladaptive changes in the sensorimotor system, resulting in performance deterioration [1,2,3], and it is known that people engaging in occupations that involve extensive repetitive movements of a specific body part get affected by task-specific dystonia [4,5]. Professional athletes who perform excessive repetitive training sometimes experience an intermittent decline in performance not caused by musculoskeletal injury; it is conventionally known as “the yips” in golfers [6], although similar problems occur in many sports [7].

Although “the yips” is common among golfers, the pathophysiology and epidemiology of the yips remain poorly understood. The yips has been considered as an anxiety-related phenomenon, with many golfers often experiencing the yips in stressful situations involving highly obsessive thoughts [8,9,10]. Sports psychologists or physiotherapists often advise players that the yips is a “misplaced focus” rather than a particular ailment [11]. In contrast, recent studies have reported that there is no indication of psychopathology in yips-affected golfers and to treat them as having focal dystonias [12,13]. Previous neurophysiological studies using electromyograms revealed a task-specific abnormal co-contraction of agonist and antagonist muscles in yips-affected golfers [14,15]. Smith et al. described the yips as a continuum between dystonia and choking (a considerable decrease in skill execution) [9], while Marquardt theorized it as a vicious cycle between a movement disorder and anxiety [16]. Based on the fact that professional golfers need excessive repetition of fine motor training for long durations to master a skilled movement (similar to that needed by musicians), these findings suggest that the yips could be considered as a type of task-specific focal dystonia [1,17,18]. In line with this notion, one previous epidemiological study revealed a significant association between longer golfing careers and the yips [8], but the findings were not consistent with another study [9].

In this study, we conduct an epidemiological investigation of the yips among highly skilled professional and non-professional golfers in Japan, to investigate the potential confounding factors associated with the yips and the effects of cultural background, since previous studies were mainly reported from Western countries. In addition, since it is known that a history of injury is a risk factor for the development of both writer’s cramp and musician’s dystonia [1,5,19], we further explore the impact of injury severity and self-treatment strategy for testing the hypothesis that the yips is a type of task-specific dystonia.

## 2. Materials and Methods

### 2.1. Standard Protocol Approvals, Registrations and Patient Consent

The ethics committee for clinical research at the Osaka University Graduate School of Medicine approved this study. All participants agreed to participate in this study and answered the questionnaire.

### 2.2. Participants

**Figure 1:**
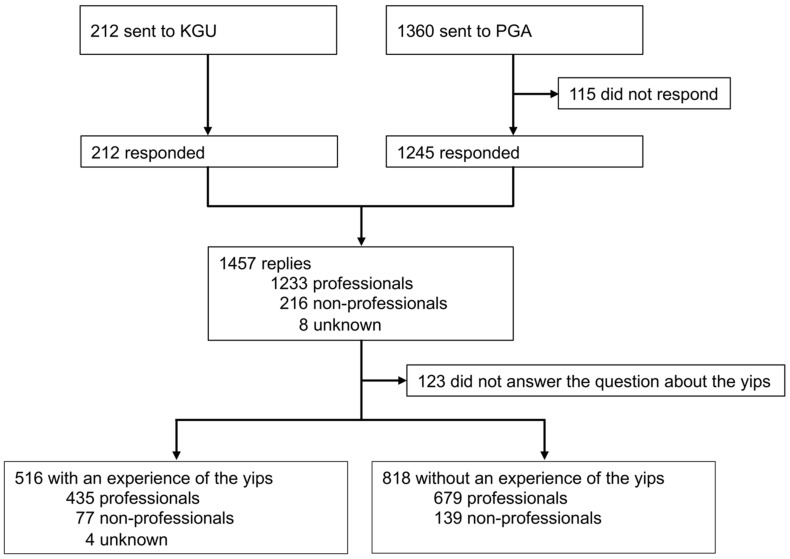
Details of distributed questionnaires. ‘Unknown’ refers to participants who did not answer the question about having professional licenses. KGU, Kansai Golf Union; PGA, Professional Golfers’ Association.

We surveyed highly skilled professional and non-professional golfers to clarify the epidemiological aspects of the yips and characterize yips-related symptoms, in collaboration with two of the largest golfers’ associations in Japan, the Professional Golfers’ Association (PGA) and the Kansai Golf Union (KGU), between June 2014 and August 2015. The PGA is the organization of golfers with a professional license in Japan; the KGU is a regional organization of highly skilled competitive amateurs. The surveys were conducted through monthly meetings and training workshops held by each association. A total of 1572 surveys were distributed to the following groups of participants: 1360 PGA members and 212 KGU members. In a training workshop conducted by the PGA, four non-professionals participated and answered the survey. Therefore, the survey involved 1356 professional and 216 non-professional golfers. Further details are shown in Figure 1.

### 2.3. Questionnaire Surveys

The questionnaire survey consisted of 28 questions, most of which were designed to be answered by checking a box. All participants answered the first 13 of the 28 questions pertaining to demographic information (age, sex and dominant hand), golfing career-related history (professional or non-professional, duration of golfing career, hours of practice per month and the annual total number of golfing rounds), the presence of musculoskeletal problems (location and degree of symptoms) and knowledge and experience of the yips. All participants received an explanation about the yips before answering the questionnaire. The questions on age, duration of golfing career, hours of practice per month and the annual total number of golfing rounds were required to be given in specific figures. When participants had musculoskeletal problems, they were requested to specify the location according to the following body parts: the neck, shoulders, upper arms, elbows, lower arms, wrists, upper back, lower back and legs. Additionally, their degree of symptoms was reported according to the following categories: mild, symptoms that neither affect golf playing nor daily living; moderate, symptoms that affect golf playing but do not influence daily living; and severe, symptoms that affect both golf playing and daily living. Multiple answers were allowed for the question on musculoskeletal problems, and if a participant responded with multiple abnormalities, the most severe symptoms were used. Participants who had experience with the yips were instructed to answer the remaining 15 questions regarding information about the yips, duration of golfing-career prior to the experience of the yips, clinical manifestations of the yips—including clubs, strokes and symptoms—and self-treatment strategies for the yips. For the question on clubs, participants were required to specify the clubs they were using when they experienced the yips (putter, iron or driver). Similarly, participants were requested to specify the situation they were in when they experienced the yips (tee shot, fairway shot, rough shot, bunker shot, approaching or putting) and the subjective symptoms suffered (jerk, spasm or tremor). With regard to the question on the self-treatment strategy for the yips, participants were asked to define the adopted strategy when they experienced the yips, according to the following categories: increased training loads, decreased training loads, changed training method and/or hitting style. If there was no appropriate option, participants wrote their strategy freely, which was grouped as “others”. Multiple answers were allowed on the items of “clubs”, “strokes”, “symptoms” and “strategy for the yips”. Our analysis explored what type of strategy led to an improvement in the yips. In this regard, the answers on “strategy for the yips” were categorized into the following groups: increasing group, participants only checked the “increased training loads”; decreasing group, participants only checked the “decreased training loads”; no change of training loads, participants checked both “increased training loads” and “decreased training loads” or did not check any of them; and others.

### 2.4. Statistical Analysis

All data were expressed as median and interquartile ranges for continuous variables and counts and as percentages for categorical variables. To compare the characteristics of participants with and without yips experience, the Wilcoxon rank-sum test for continuous variables and the chi-square test for categorical variables were performed.

To identify factors associated with the experience of the yips, the multivariate logistic regression model was used with the inclusion of professional or non-professional golfing careers, hours of practice per month, the annual total number of golfing rounds and the severity of musculoskeletal problems as explanatory variables. Non-linear restricted cubic splines were used to assess the non-linearity of all the continuous covariates. Missing data were imputed using multiple imputation methods with the “areg.impute” function in the rms package of R [20].

To investigate the association between musculoskeletal problems and the yips in detail, multivariate logistic regression analysis was conducted with the cross-product term between the severity of the musculoskeletal problem and the injured part of the body, which was divided into the upper body (the neck, shoulders, upper arms, elbows, lower arms, wrists and upper back) and lower body (the lower back and legs). This analysis was performed using data including only professional players because the information on the injured body part was collected only from the professional players.

To examine the factors associated with the degree of improvement of the yips (worse = 1, no change = 2, improvement = 3), a proportional odds ordinal logistic regression model with variables indicating adopted self-treatment strategy for the yips (changing a training method and/or hitting style, increase and/or decrease in training loads and the presence or absence of other training methods) as explanatory variables was performed among the participants who had experienced the yips. The effects of the training strategy differed between professional and non-professional players based on their a priori chosen relevance. The interaction term between a variable indicating a professional player and the dummy variables indicating the training strategy was assessed, with the inclusion of their cross-product terms in the multivariate regression, along with the main effect variables. The global test for all interaction terms was first assessed after detecting its statistical significance. The statistical significance of each interaction term was assessed. The interaction terms with statistical significance were then included in the final model. Adjustments and missing data imputations were made similar to those in the binary logistic regression model.

All statistical inferences were made with two-sided analysis at the 5% significance level, except for the interaction analyses. Because of the underpowered nature of an interaction analysis, a two-sided significance level of 20% was used for all interactions [21]. All statistical analyses were performed with R software using the rms package [20].

## 3. Results

### 3.1. Demographics of Respondent Golfers

The demographics of the participants who responded are presented in Table 1. A total of 92% responded, of which 85% were professionals, 96% were men and 96% were right-handed. The median age, duration of golfing careers, hours of practice per month and annual total numbers of golfing rounds were 47 years, 28 years, 15 h and 20 rounds, respectively. Musculoskeletal problems were described by 47.4% of respondents, of which 26% were mild, 11% were moderate and 9% were severe. Low back pain (46%) was the most common symptom in respondents with musculoskeletal problems (Supplemental Table S1). Most golfers, 98% of respondents, had knowledge of the yips.

### 3.2. Comparison of Golfers with and without an Experience of the Yips

**Figure 2:**
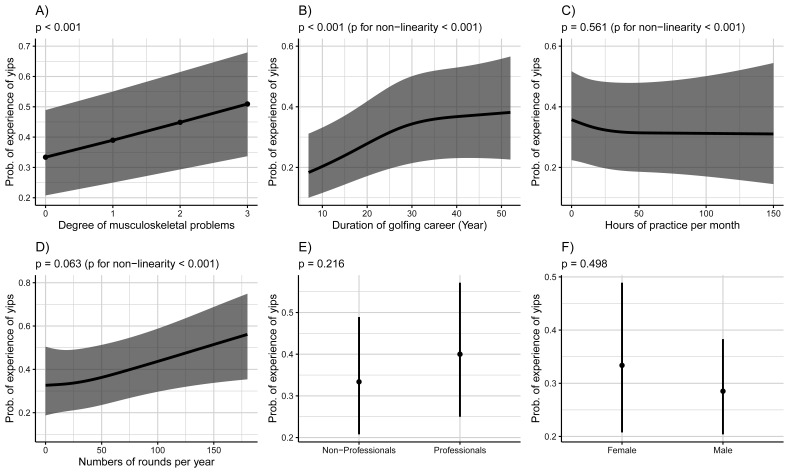
Analysis of the effects of different factors on yips experience. Association of the yips experience and musculoskeletal problems (**A**), golfing career (**B**), hours of practice per month (**C**), numbers of rounds per year (**D**), having a professional license (**E**) and gender (**F**).

The characteristics of golfers with and without the experience of the yips are presented in Table 2. Approximately 39% of golfers experienced the yips. Golfers with an experience of the yips were older, had longer golfing careers and suffered from musculoskeletal problems more often than those without an experience of the yips. The factors associated with the experience of the yips are shown in Figure 2. The multivariate non-linear regression analysis revealed that having musculoskeletal problems (Figure 2A, *p* < 0.001) and a longer golfing career (Figure 2B, *p* < 0.001) were independent factors associated with the experience of the yips. Specifically, more severe musculoskeletal problems were associated with higher odds of having the yips (Figure 2A).

**Figure 3:**
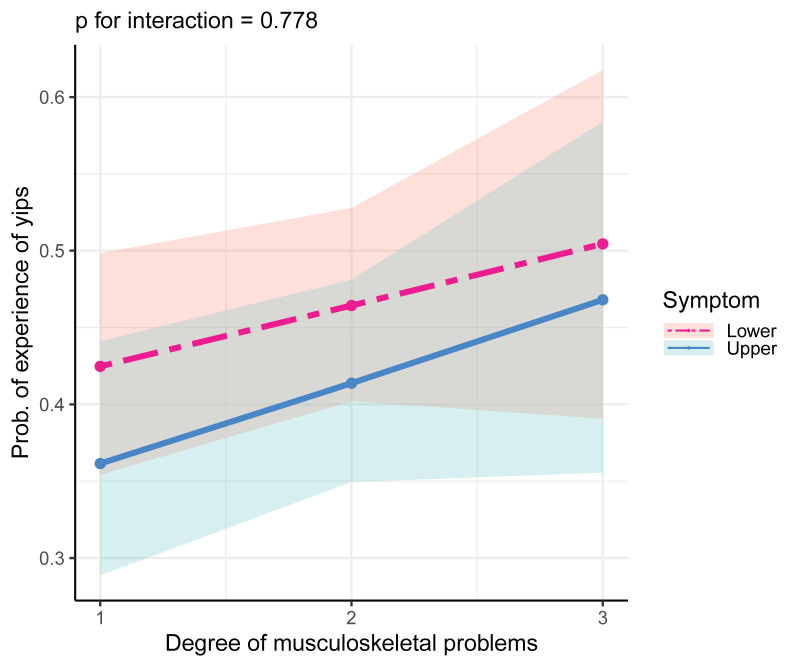
The impact of musculoskeletal symptoms with respect to the upper and lower body on the yips experience. The analysis revealed that there was no interaction between the upper and lower body’s musculoskeletal problems.

To examine in detail musculoskeletal symptoms and the experience of the yips, musculoskeletal symptoms were differentiated based on the upper and lower body and their effects were investigated. The results showed no effect modification by the injured body parts (Figure 3).

### 3.3. Clinical Manifestations of the Yips

The clubs used by golfers when they experienced the yips were putters (54%), drivers (31%) and irons (19%), respectively. The situations during which the yips occurred were putting (54%), approaching (43%), tee shot (33%), fairway shot (14%), bunker shot (8%) and rough shot (7%). The subjective symptoms were spasm (29%), jerk (23%) and tremor (15%). The adopted self-treatment strategies were increased training loads (33%), decreased training loads (10%), changed training method and/or hitting style (63%) and others (20%) (Supplemental Table S2). Only 8% of the golfers received treatment in a hospital.

### 3.4. Factors Associated with Improvement of the Yips

**Figure 4:**
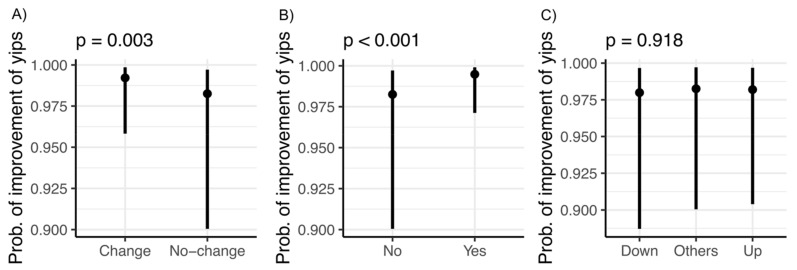
Proportional odds logistic regression analysis of the factors associated with the improvement of the yips. (**A**) Changing the training method and/or hitting style; (**B**) “other” strategy; (**C**) decreasing and/or increasing the amount of practice.

The results of the proportional odds logistic regression analysis are shown in Figure 4. When golfers experienced the yips, changing the training method and/or hitting style (Figure 4A, *p* = 0.003) and using an “other” strategy (Figure 4B, *p* < 0.001) were associated with an improvement in the yips. There was no statistical relationship between increasing or decreasing the amount of practice and improvement in the yips (Figure 4C, *p* = 0.92). The analysis revealed that there was an interaction between the professional golfers and taking an “other” strategy (Supplemental Table S3).

## 4. Discussion

Although there are several epidemiological studies on the prevalence of the yips in highly skilled golfers, those studies have the limitation that the response rate was less than half of the distributed questionnaires [9,10]. In contrast, our study contains a total of 1457 completed surveys with an exceptionally high response rate of 92%. To our knowledge, this is the largest study to date to examine the yips among golfers, and the large sample size with a high response rate is one of the most vital aspects of this study.

Our findings revealed that 39% of the golfers experienced the yips, confirming the findings of previous reports with 22–48% of golfers experiencing the yips in their careers [9,10,22]. As evidenced by a high percentage of respondents who were aware of the yips (98%) in our study, it is undeniably a well-known condition among golfers’ circles. Often, golfers self-diagnose their musculoskeletal problem to a yips-like phenomenon. In our study, more golfers with an experience of the yips had musculoskeletal problems (56%) than those without (42%). Musculoskeletal problems were most likely to occur in the lower back, leg or shoulder. Although it is reasonable for participants to self-diagnose their musculoskeletal symptoms as the yips, we believe that the possibility of such practice having the results presented in this study is unlikely as we conducted the questionnaire after explaining the yips to the respondents.

As reported in earlier studies, our survey also revealed that putting was the most common stroke, and jerking was the most common symptom in golfers who experienced the yips. Our study confirmed that the yips are a common problem among highly-skilled golfers, and cultural and racial backgrounds did not affect the yips phenomenon.

In our study, the golfers who experienced the yips were older and had longer golfing careers than those without the yips. Furthermore, a longer golfing career was the independent factor associated with yips experience. Considering that increased workloads are known to be one of the risk factors for the development of task-specific focal dystonia [5,19], it is reasonable to consider that extensive repetition of fine motor control for longer durations is associated with yips experience. In addition, the beneficial effect of changing the training method and/or hitting style suggests a similar pathophysiology to the writer’s cramp, in which changing the pen-grip provides an immediate beneficial effect [23]. Whether the yips have a neurological or psychological etiology is still debatable, but our findings support the hypothesis that the yips could be a type of task-specific focal dystonia similar to the writer’s cramp or musician’s dystonia [1,2,5,24].

In addition, it was observed in our study that the golfers with yips experience more often had musculoskeletal problems than those without. Ordered logistic regression analysis showed that having musculoskeletal problems was an independent factor related to yips experience, and more severe musculoskeletal symptoms were associated with higher odds of having the yips. A previous study suggested that ulnar neuropathy can initiate specific dystonia by inducing a central disorder of motor control [25]. It is supposed that musculoskeletal problems could lead to the disturbance of the afferent input to the central nervous system and cause disorganization of the motor control system.

Our study has several limitations. First, because of the cross-sectional nature of this study, our results can only suggest a relationship between the yips and various confounding factors, including musculoskeletal problems and golfing career, but cannot infer causality. To clarify the temporal relationship between musculoskeletal problems and the yips, a prospective analysis of the study is warranted. Second, our survey consisted of a self-reporting questionnaire; this might have overestimated the actual number of golfers experiencing the yips [22]. Among musicians, approximately 1% of all professional practitioners are estimated to have task-specific dystonia [19]. In comparison, the prevalence of golfer’s yips in this study and previous reports is substantially higher, and may possibly be contaminated by various cases of “yips-like” phenomena. To clarify the actual prevalence of the yips, further studies with neurophysiological confirmation are needed. The yips are a peculiar condition involving both neurological and psychological features, which are not only appropriate for investigation in anatomical and functional studies but also in studies involving the dynamic networks of brain connectivity.

## 5. Conclusions

The yips are a common problem among highly skilled professional and non-professional golfers, regardless of their cultural and racial backgrounds. Repetition of fine motor control for long durations and musculoskeletal problems are associated with yips experience, suggesting that the yips have similar characteristics to task-specific movement disorders, such as writer’s cramp and musician’s dystonia. Although further studies are needed to validate our findings, this study provides clues for understanding the pathophysiology of the yips as a type of task-specific dystonia.

## Figures and Tables

**Table 1:** Demographics of the golfers who responded.

Variable	Participants(n)	
Age (years), median (IQR)	1454	47 (40–54)
Professionals, % (n)	1449	85 (1233)
Male, % (n)	1455	96 (1399)
Dominant hand, % (n)	1447	
Both		0 (3)
Left		4 (54)
Right		96 (1390)
Duration of golfing career (years), median (IQR)	1445	28 (21–35)
Monthly practice (hours), median (IQR)	1435	15 (6–30)
Annual total golfing rounds (number), median (IQR)	1404	20 (10–40)
Musculoskeletal problems, % (n)	1454	47 (689)
Degree of musculoskeletal symptoms, % (n)	1419	
None		54 (767)
Mild		26 (362)
Moderate		11 (158)
Severe		9 (132)
Knowledge of the yips, % (n)	1385	98 (1362)

Not all respondents answered all the survey questions; therefore, the numbers of cases and percentages do not match. Percentages have been rounded up for simplicity of presentation and might not total 100% in all cases. IQR, interquartile range.

**Table 2:** Comparison between golfers with and without the yips experience.

	With the Yips Experience (n = 516)	Without the Yips Experience (n = 818)	*p* Value
Age (years), median (IQR)	48 (42–55)	47 (41–53)	0.027
Professionals, % (n)	85 (435)	83 (679)	0.44
Male, % (n)	96 (496)	96 (781)	0.63
Dominant hand, % (n)			<0.14
Both	0 (2)	0 (0)	
Left	3 (17)	4 (35)	
Right	96 (496)	96 (777)	
Duration of golfing career (years), median (IQR)	30 (23–37)	27 (20–35)	<0.001
Monthly practice (hours), median (IQR)	15 (8–30)	15 (6.4–30)	0.70
Annual total golfing rounds (number), median (IQR)	20 (10–50)	20 (10–40)	0.36
Musculoskeletal problems, % (n)	56 (290)	42 (347)	<0.001
Degree of musculoskeletal symptoms, % (n)			<0.001
None	44 (225)	58 (472)	
Mild	30 (150)	24 (195)	
Moderate	13 (68)	10 (82)	
Severe	13 (64)	8 (61)	

Not all respondents answered all the survey questions; therefore, the numbers of cases and percentages do not match. Percentages have been rounded up for simplicity of presentation.

## Data Availability

The data presented in this study are available on request from the corresponding author. The data are not publicly available due to privacy or ethical restrictions.

## Supplementary Materials

The following are available online at https://www.mdpi.com/article/10.3390/sports9060071/s1: Table S1: Characteristics of musculoskeletal problems, Table S2: Clinical manifestation of the yips and Table S3: Factors associated with improvement in the yips (results of interaction analysis).
