# Supplementary material for: Association of the Yips and Musculoskeletal Problems in Highly Skilled Golfers: A Large Scale Epidemiological Study in Japan
Source: Sports (Basel). 2021 May 21;9(6):71. doi: 10.3390/sports9060071 (PMC8224395; doi:10.3390/sports9060071)
Supplement: Supplementary file 1 [file sports-09-00071-s001.zip › sports-1178736-supplementary.pdf]

sports

MDPI

Supplementary Materials

Association of the Yips and Musculoskeletal Problems in Highly Skilled Golfers: A Large Scale Epidemiological Study in Japan

Yasufumi Gon [1,*], Daijiro Kabata [2], Sadahito Kawamura [3,4], Masahito Mihara [5], Ayumi Shintani [2], Ken Nakata [3] and Hideki Mochizuki [1]

[1] Department of Neurology, Graduate School of Medicine, Osaka University, 2-2 Yamada-oka, Suita, Osaka 565-0871, Japan; hmochizuki@neurol.med.osaka-u.ac.jp
[2] Department of Medical Statistics, Graduate School of Medicine, Osaka City University, 1-4-3 Asahi-machi, Osaka Abeno-ku, Osaka 545-8586, Japan; kabata.daijiro@med.osaka-cu.ac.jp (D.K.); ayumi.shintani@gmail.com (A.S.)
[3] Department of Medicine for Sports and Performing Arts, Graduate School of Medicine, Osaka University, 2-2 Yamada-oka, Suita, Osaka 565-0871, Japan; kawamura@bc.iij4u.or.jp (S.K.); ken.nakata7@gmail.com (K.N.)
[4] Kawamura Orthopedic Clinic, 1-10-30 Misaki, Osaka minato-ku, Osaka 552-0016, Japan
[5] Department of Neurology, Kawasaki Medical University, 557 Matsushima, Kurashiki, Okayama 701-0192, Japan; mihara@med.kawasaki-m.ac.jp
* Correspondence: gon@neurol.med.osaka-u.ac.jp; tel.: +81-6-6879-3571

Citation: Gon, Y.; Kabata, D.; Kawamura, S.; Mihara, M.; Shintani, A.; Nakata, K.; Mochizuki, H. Association of the Yips and Musculoskeletal Problems in Highly Skilled Golfers: A Large Scale Epidemiological Study in Japan. *Sports* **2021**, *9*, 71. https://doi.org/10.3390/sports9060071

Academic Editor: Silvio Lorenzetti

Received: 26 March 2021
Accepted: 18 May 2021
Published: 21 May 2021

Publisher's Note: MDPI stays neutral with regard to jurisdictional claims in published maps and institutional affiliations.

Copyright: © 2021 by the authors. Submitted for possible open access publication under the terms and conditions of the Creative Commons Attribution (CC BY) license (http://creativecommons.org/licenses/by/4.0/).

Abstract: The yips are a set of conditions associated with intermittent motor disturbances that affect precision movement, especially in sports. Specifically, skilled golfers suffer from the yips, although its clinical characteristics and pathophysiology have not been well-studied. We survey skilled golfers to characterize their yips-related symptoms, to explore potential confounding factors associated with the yips. Golfers' demographic information, golfing-career-related history, musculoskeletal status and manifestations of the yips are surveyed. Among the 1576 questionnaires distributed, 1457 (92%) responses are received, of which 39% of golfers have experienced the yips. The median age and golfing careers are 48 and 28 years, respectively. Golfers who have experienced the yips are older and have had longer golfing careers and more frequent musculoskeletal problems than those without experience of the yips. The multivariate logistic regression analysis reveals that a longer golfing career and musculoskeletal problems are independent factors associated with yips experience. More severe musculoskeletal problems are associated with higher odds of experiencing the yips. A positive association between the yips and musculoskeletal problems is also observed. The yips have similar characteristics to task-specific movement disorders, with a detrimental effect caused by excessive repetition of a routine task. These findings support the notion that the yips are a type of task-specific dystonia.

Keywords: yips; golfing career; musculoskeletal symptoms; task-specific dystonia

Table S1. Characteristics of musculoskeletal problems.

**Location**	Musculoskeletal problems, n = 689	Degree of musculoskeletal symptoms			
		Mild	Moderate	Severe	Unknown[†]
**Neck**	106 (15)	62 (58)	17 (16)	23 (22)	4 (4)
Shoulder or upper arm	149 (22)	83 (56)	35 (23)	25 (17)	6 (4)
Elbow	113 (16)	70 (62)	23 (20)	16 (14)	4 (4)
Lower arm	13 (2)	4 (31)	3 (23)	6 (46)	0 (0)
Wrist	120 (17)	61 (51)	31 (26)	19 (16)	9 (8)
Upper back	53 (8)	28 (53)	12 (23)	10 (19)	3 (6)
Lower back	314 (46)	171 (54)	82 (26)	47 (15)	14 (4)
Leg	219 (32)	106 (48)	50 (23)	48 (22)	15 (7)
Unknown[†]	57 (8)	41 (72)	14 (25)	0 (0)	2 (4)

All data are shown in counts and percentages. Percentages have been rounded up for simplicity of presentation and may not equal 100% in all cases.

Table S2. Clinical manifestation of the yips.

Variable	
Affected club	
Putter	281 (54)
Iron	98 (19)
Driver	158 (31)
Affected stroke	
Putting	279 (54)
Approaching	224 (43)
Bunker shot	40 (8)
Rough shot	34 (7)
Fairway shot	72 (14)
Tee shot	168 (33)
Symptoms	
Spasm	151 (29)
Jerk	117 (23)
Tremor	75 (15)
Strategy for the yips	
Increasing training loads	139 (33)
Decreasing training loads	53 (10)
Changing training methods and/or hitting style	327 (63)
Others[†]	101 (20)

All data are shown in counts and percentages. Percentages have been rounded up for simplicity of presentation and may not equal 100% in all cases. [†]Others refer to strategies such as changing of preshot routine and/or thought.

Table S3. Factors associated with improvement in the yips (results of interaction analysis).

**Characteristic**	**Odds ratio**	**95% confidence interval**	***p* value**
Amateur and other strategy	1.24	0.38–4.01	0.72
Professional and other strategy	4.50	2.28–8.86	<0.001
Professional*other strategy			0.05

Only the result of interaction analysis between professional/amateur and other strategy was shown in this table because we found no other significant relationships.

.
